# Preparation and Crystal Structure of a Rhenium Analogue of the Cationic Renal Agent, Tc-99m Diaminocyclohexane

**DOI:** 10.1155/MBD.2000.141

**Published:** 2000

**Authors:** Luigi G. Marzilli, Lory Hansen, Andrew Taylor, Rene Lachicotte

**Affiliations:** 1 Department of Chemistry Emory University Atlanta GA 30322 USA; 2 Department of Radiology Emory University Atlanta GA 30322 USA; 3 Department of Chemistry University of Rochester NY 14627 USA

## Abstract

We report here a chemical study on a Re analogue of one of the few cationic Tc-99m tracers previously investigated as an agent for effective renal plasma flow (ERPF) measurement. Cationic Tc-99m tracers have the potential for overcoming problems associated with common anionic Tc-99m tracers in patients who have developed a uremic state. The Tc-99m-DACH tracer, prepared from 1,2-diaminocyclohexane (1,2-DACH), is the only cationic renal agent tested in humans and has seven possible isomers. The complex isolated from the reaction of the racemic mixture, (±)-*trans*-1,2-DACH, and ReIO_2_(PPh_3_)_2_ after conversion to the BPh_4_^-^ salt was found by X-ray crystallography to be the *meso* isomer, *trans*-[ReO_2_ (*trans-R,R*-1,2-DACH)(*trans-S,S*-l,2-DACH)][BPh_4_]·MeOH·2H_2_O (**1**). The structural parameters for **1** are normal. The complex is highly symmetrical, suggesting that the analogous *meso* Tc-99m-DACH agent is also symmetrical. Studies of other Tc-99m-DACH agents that were made from *cis*-1,2-DACH or individual *trans*-1,2-DACH enantiomers show that the biodistribution is not very dependent on the starting 1,2-DACH ligand stereochemistry; these agents must be less symmetrical than the *meso* Tc-99m-DACH agent analogue of **1**. Thus, the overall charge and lipophilicity (similar for all Tc-99m-DACH isomers) exert a greater influence on biodistribution than the specific structural features of the different Tc-99m-DACH isomers.


### PREPARATION AND CRYSTAL STRUCTURE OF A RHENIUM ANALOGUE OF THE CATIONIC RENAL AGENT, Tc-99m DIAMINOCYCLOHEXANE

Luigi G. Marzilli*[1], Lory Hansen[2], Andrew Taylor[2], and Rene Lachicotte[3]

[1]Department of Chemistry, Emory University, Atlanta, GA 30322 USA <lmarzil@emory.edu>
[2]Department of Radiology, Emory University, Atlanta, GA 30322 USA
[3]Department of Chemistry, University of Rochester, NY 14627 USA

### ABSTRACT

We report here a chemical study on a Re analogue of one of the few cationic Tc-99m tracers previously investigated as an agent for effective renal plasma flow (ERPF) measurement. Cationic Tc-99m tracers have the potential for overcoming problems associated with common anionic Tc-99m tracers in patients who have developed a uremic state. The Tc-99m-DACH tracer, prepared from 1,2-diaminocyclohexane (1,2-DACH), is the only cationic renal agent tested in humans and has seven possible isomers. The complex isolated from the reaction of the racemic mixture, (±)-*trans*-1,2-DACH, and $ReIO_2(PPh_3)_2$ after conversion to the $BPh_4^-$ salt was found by X-ray crystallography to be the *meso* isomer, *trans*-[$ReO_2$(*trans-R,R*-1,2-DACH)(*trans-S,S*-1,2-DACH)][$BPh_4$]•MeOH•2$H_2O$ (**1**). The structural parameters for **1** are normal. The complex is highly symmetrical, suggesting that the analogous *meso* Tc-99m-DACH agent is also symmetrical. Studies of other Tc-99m-DACH agents that were made from *cis*-1,2-DACH or individual *trans*-1,2-DACH enantiomers show that the biodistribution is not very dependent on the starting 1,2-DACH ligand stereochemistry; these agents must be less symmetrical than the *meso* Tc-99m-DACH agent analogue of **1**. Thus, the overall charge and lipophilicity (similar for all Tc-99m-DACH isomers) exert a greater influence on biodistribution than the specific structural features of the different Tc-99m-DACH isomers.

### INTRODUCTION

A simple and accurate measurement of effective renal plasma flow (ERPF) could lead to improved diagnosis and management of patients with pre-renal azotemia and for the development of better strategies for prevention and reversal of renal failure. With the development of the uremic state, there is a progressive buildup of organic anions in plasma.[1-4] These accumulated anions can competitively inhibit renal tubular transport, reduce the clearance of anionic substrates, and lead to variable or spuriously low measurements of ERPF and tubular function.[1, 2, 4-8] Most studies directed at finding a superior renal agent have focused on anionic Tc-99m agents,[9, 10] but these must have an extraordinarily high affinity for the tubular transport receptor to avoid the problem of competitive inhibition by organic anions in plasma. Evidence exists that this problem can be avoided, however, by the development of a cationic tracer for ERPF measurement. C-14 tetraethylammonium cation (TEA), one of the earliest cationic agents investigated is secreted by the renal tubular organic base transport mechanism.[11] Studies in azotemic dogs demonstrated that the extraction fraction of TEA was significantly higher than that of the "gold standard" para-aminohippurate (PAH) anion; in addition, unlike PAH, the extraction of TEA was independent of the level of blood urea nitrogen.[2] On the basis of these studies, the investigators concluded that TEA is superior to PAH for the evaluation of the renal circulation in azotemia. The most useful radioisotope for radiopharmaceuticals is $^{99m}$Tc.

For these reasons, we have initiated our research program aimed at finding clinically useful cationic renal agents with a chemical study of Tc-99m-DACH, the only Tc-99m cationic renal agent for which studies in humans have been reported.[12] Tc-99m-DACH, the product of labeling 1,2-diaminocyclohexane (1,2-DACH),[13, 14] is non-toxic, only 20% protein bound, and is cleared more rapidly than tracers measuring the glomerular filtration rate (GFR); therefore, transport of Tc-99m-DACH into the urine must be through the tubules. The clearance of Tc-99m DACH can be competitively blocked using thiamine as a cationic competitor, confirming cationic tubular transport; infusion of thiamine has no effect on tracers used to measure glomerular filtration or on the tubular transport of anionic tracers.[12, 15] The clearance of Tc-99m-DACH is 80% of that of OIH in the mouse but only 30% of that of OIH in healthy human volunteers.[13, 15] The chemical form of the Tc-99m-DACH agent studied by Solanki and others is not well characterized and may contain a single or a mixture of isomeric forms. The 1,2-DACH ligand has three isomers: one *cis* isomer (with *R* and *S* carbons and thus the *meso* form) and two *trans* isomers (normally available as a racemic mixture of *R,R* and *S,S* enantiomers). The Tc-99m-DACH agent likely contains two 1,2-DACH ligands and can be formulated as *trans*-[$TcO_2$(1,2-DACH)$_2$]$^+$. Thus, seven different Tc-99m-DACH isomers are possible with a *trans* $TcO_2^+$ core (Figure 1). Different isomeric forms of anionic renal tracers may have widely different clearances,[16, 17] and, by analogy, different isomeric forms of cationic renal tracers may also have very different clearances. In fact, one of the Tc-99m-DACH isomers could have a high clearance in humans but that clearance could be obscured by the relatively low clearances of the other isomers in a mixture. To test this possibility, we previously compared renal excretion and biodistribution in Sprague-Dawley rats of Tc-99m-*cis*-1,2-DACH, Tc-99m-*trans-R,R*-1,2-DACH, Tc-99m-*trans-S,S*-DACH and a Tc-99m-*trans*-(±)-1,2-DACH isomeric mixture.[18] This mixture was found by HPLC to contain the statistically expected mixture of 25% Tc-99m-*trans-S,S*-1,2-DACH, 25% Tc-99m-*trans-R,R*-1,2-DACH, and 50% *meso*-Tc-99m-(*trans-R,R-/S,S*-1,2-DACH). Little difference was found, however, in the rate of renal excretion or biodistribution of the isomers.

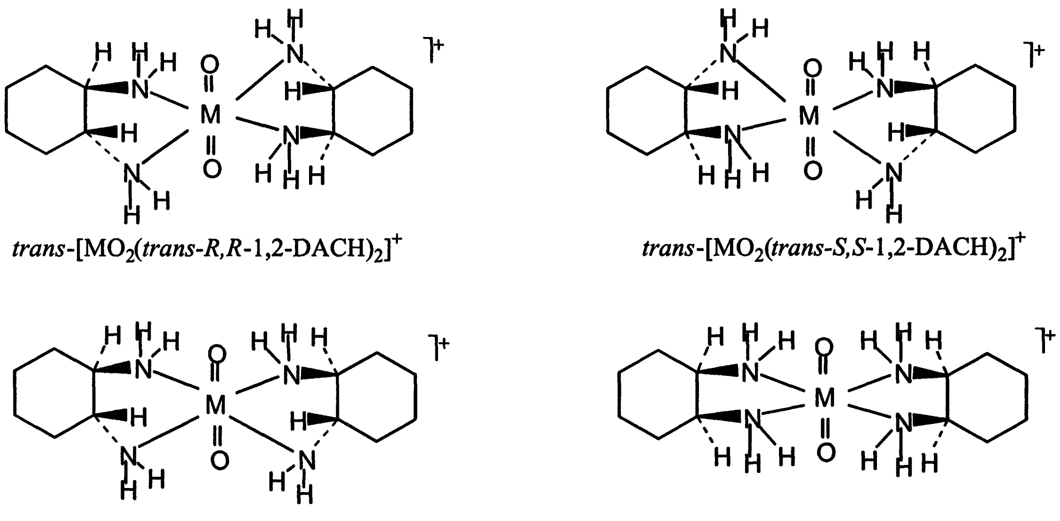

*trans*-$[MO_2($*trans-R,R*-1,2-DACH$)_2]^+$

*trans*-$[MO_2($*trans-S,S*-1,2-DACH$)_2]^+$

*trans*-$[MO_2($*trans-R,R*-1,2-DACH)(*trans-S,S*-1,2-DACH$)]^+$
*meso* isomer

*trans*-$[MO_2($*cis*-1,2-DACH$)_2]^+$

**Figure 1.** Schematic drawing of some possible isomers of *trans*-$[MO_2(1,2\text{-DACH})_2]^+$ cations; isomers in which *cis*- and *trans*-1,2-DACH are within the same complex are not shown. One possible isomer of the complex with the *cis*-1,2-DACH ligand has both cyclohexane rings pointing in the same direction (shown); the other isomer, with rings pointing away from each other to either side of the coordination plane, is not shown.

An important aspect of Tc-99m radiopharmaceutical development is the study of the Re analogues, which typically have nearly identical structures to the Tc counterpart and which are environmentally more desirable compounds for chemical study. In this study, we determined the crystal structure of a Re analogue of one *trans*-$[TcO_2(1,2\text{-DACH})_2]X$ isomer so that we could begin to identify the specific structural features associated with rapid cationic renal transport.

### MATERIALS AND METHODS

$ReIO_2(PPh_3)_2$ was prepared according to the literature procedure.[19] The racemic mixture of ligands, (±)-*trans*-1,2-diaminocyclohexane ((±)-*trans*-1,2-DACH) was obtained from Aldrich. Elemental analyses were performed by Atlantic Microlabs, Atlanta, GA. The IR spectrum (KBr pellet) was recorded on a Nicolet 510M instrument.

**Synthesis.** ***trans*-[ReO$_2$(*trans*-1,2-DACH)$_2$]I•H$_2$O.** $ReO_2I(PPh_3)_2$ (0.43 g, 0.5 mmol) was suspended in MeOH (5 mL); (±)-*trans*-1,2-DACH (0.23 g, 2.0 mmol) was added, and the reaction mixture was heated at reflux for 30 min. After the solution was cooled to room temperature, the pale yellow precipitate that formed was collected, washed with chilled MeOH, and air dried. Yield, 0.13 g (45%). An analytical sample was prepared by recrystallization from MeOH. Anal. Calcd for the monohydrate $C_{12}H_{30}IN_4O_3Re$: C, 24.37; H, 5.11; N, 9.47. Found: C, 24.52; H, 5.10; N, 9.20.

***trans*-[ReO$_2$(*trans*-1,2-DACH)$_2$][BPh$_4$]•MeOH•2H$_2$O (1).** The iodide salt (0.11 g, 0.2 mmol) and $Na[BPh_4]$ (0.07 g, 0.2 mmol) were dissolved in MeOH (10 mL). Yellow crystals of *trans*-$[ReO_2($*trans*-1,2-diaminocyclohexane$)_2][BPh_4]$•MeOH•$2H_2O$, deposited after addition of $H_2O$ to a faint cloudiness, were collected and air dried. X-ray quality crystals were obtained from the filtrate following collection of the first crop of microcrystals. Yield, 98 mg (59%). Anal. Calcd for the monohydrate $C_{37}H_{56}BN_4O_5Re$: C, 53.29; H, 6.77; N, 6.72. Found: C, 53.87; H, 6.72; N, 6.79. FTIR: Spectrum in the 550 to 1200 $cm^{-1}$ range of **1** was nearly identical to that of Na[$BPh_4$], except for a strong band at 808 $cm^{-1}$, which is assigned to $\nu_{Re=O}$. The $^1H$ NMR spectra of all *trans*-[$ReO_2$(1,2-DACH$)_2$][$BPh_4$] isomers examined in DMSO-$d_6$ are relatively uninformative, containing broad peaks for the 1,2-DACH ligand in the range ~1-2 ppm and for the [$BPh_4$]$^-$ counterion in the range 6.7-7.4 ppm.

**X-ray Crystallography.** An orange crystal of **1** of approximate dimensions 0.28 x 0.22 x 0.22 $mm^3$ was mounted under Paratone-8277 onto a glass fiber, and immediately placed in a cold nitrogen stream at –80 °C on the X-ray diffractometer. X-ray intensity data were collected on a standard Siemens SMART CCD Area Detector System equipped with a normal focus molybdenum-target X-ray tube operated at 2.0 kW (50 kV, 40 mA). Data (1321 frames, 1.3 hemispheres) were collected using a narrow frame method with scan widths of 0.3° in ω and exposure times of 10 s/frame using a detector-to-crystal distance of 5.09 cm (maximum $2\theta$ angle of 56.6°). The total data collection time was approximately 6 h. Frames were integrated to a maximum $2\theta$ angle of 56.3° with the Siemens SAINT program to yield a total of 21389 reflections, of which 8364 were independent ($R_{int}$ = 1.77%, $R_{sig}$ = 2.34%) and 7117 were above 2σ(I). Laue symmetry revealed a monoclinic crystal system, and the final unit cell parameters were determined from the least-squares refinement of three-dimensional centroids of 8192 reflections. Data were corrected for absorption with the SADABS program.[20]

The space group was assigned as *I2/a*, and the structure was solved by using direct methods and refined employing full-matrix least-squares on $F^2$ (Siemens SHELXTL, version 5.04). The structures refined to a goodness of fit of 1.009 and final residuals of $R_1$ = 3.30% [I>2σ(I)], $wR_2$ = 8.42% [I>2σ(I)].

### RESULTS AND DISCUSSION

$^{99m}$Tc radiopharmaceuticals are characterized chemically by preparing $^{99}$Tc and Re derivatives. $^{99}$Tc derivatives provide the most representative chemical data for $^{99m}$Tc radiopharmaceuticals. However, since the half-life of $^{99}$Tc is ~200,000 years, the isotope poses a serious contamination hazard. $^{99}$Tc and Re complexes have essentially identical physical properties and hence identical structures. Naturally occurring Re is non-radioactive and environmentally preferable. Since our aim was to understand the structure of Tc-99m-DACH isomers, we prepared a Re derivative. Ligand exchange of $ReIO_2(PPh_3)_2$ with (±)-*trans*-1,2-DACH proceeded cleanly with an excess (4 equiv) of diamine. Conversion of the $I^-$ salt to the [$BPh_4$]$^-$ salt aided crystallization of the *trans*-[$ReO_2$(*trans*-1,2-DACH$)_2$]$^+$ cation for the X-ray diffraction study.

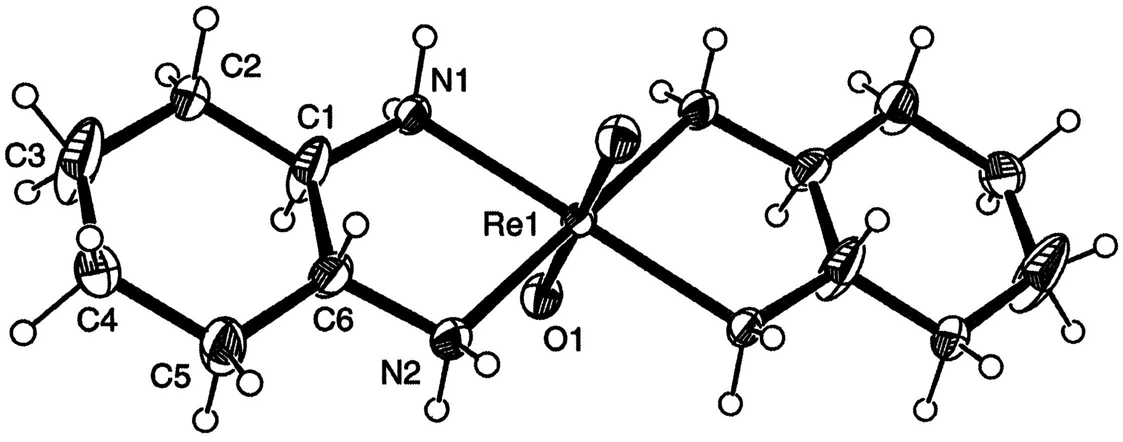

**Figure 2.** Perspective drawing of cation #1 of *trans*-[$ReO_2$(*trans-R,R*-1,2-DACH)(*trans-S,S*-1,2-DACH)][$BPh_4$]•MeOH•2$H_2O$ (**1**) with 50% probability for the thermal ellipsoids.

**X-ray Crystallography.** The single crystal selected for the X-ray diffraction study consisted of the *meso* isomer (**1**) with one *trans-R,R-* and one *trans-S,S*-1,2-DACH ligand. Crystal data and refinement parameters are presented in Table 1. The asymmetric unit of **1** consists of two independent cations. For each cation the metal atom is located at a special position (inversion center, occupancy one half); the second half of each cation is generated by the symmetry operations -x+3/2,-y+1/2,-z+1/2 (cation #1) and -x,-y,-z (cation #2). Differences in bond distances and angles within the inner coordination sphere of the two cations were insignificant (Table 2). Figure 2 shows a perspective drawing of cation #1. Selected bond distances and angles for both cations are listed in Table 2.

The cation of **1** is a *trans*-dioxo complex with two 1,2-DACH ligands coordinated in the equatorial plane. The Re-N and Re-O bond distances are normal.[21] Bond angles within the inner coordination sphere correspond to a regular octahedron within 1°, except that the bite angles of the DACH ligands are acute

(79°), while the inter-ligand N-Re-N bond angles are obtuse (101°). The 1,2-DACH ligands adopt a chair conformation. In cation #1, the conformation of the 1,2-DACH ligands is flattened, with torsion angles ranging from 21-26°, compared to the 56° values obtained for cyclohexane in the gas phase.[22] For cation #2, the torsion angles within the cyclohexane ring were larger (38-51°) but still smaller than observed for the uncoordinated ligand.

**Table 1.** Crystal Data for *trans*-[$ReO_2$(*trans-R,R*-1,2-DACH)(*trans-S,S*-1,2-DACH)][$BPh_4$]•MeOH•$2H_2O$ (**1**)

| | |
|---|---|
| chemical formula | $C_{37}H_{52}BN_4O_5Re$ |
| fw | 829.84 |
| $T$(K) | 193 (2) |
| λ (Å) | 0.71073 |
| space group | $I2/a$ |
| unit cell dimensions | $a$ = 18.77350 (10) Å |
| | $b$ = 20.7075 (3) Å |
| | $c$ = 19.1457 (3) Å |
| | $\beta$ = 99.2170 (10) deg |
| $V$ (Å)$^3$ | 7346.8 (2) |
| $Z$ | 8 |
| $\rho_{calcd}$ (g cm$^{-3}$) | 1.500 |
| absorption coefficient | 3.354 mm$^{-1}$ |
| $R$ indices [$I > 2\sigma(I)$][a] | $R_1$ = 0.0330, $wR_2$ = 0.0842 |
| $R$ indices (all data)[a] | $R_1$ = 0.0409, $wR_2$ = 0.0884 |
| Deposition number | CCDC 145438 |

[a] $R_1 = (\Sigma||F_o| - |F_c||)/\Sigma|F_o|$ and $wR_2 = [\Sigma[w(F_o^2 - F_c^2)^2]/\Sigma[w(F_o^2)^2]]^{1/2}$, where $w = 1/[\sigma^2(F_o^2) + (aP)^2 + bP]$ and $P = [(\max;0,F_o^2) + 2F_c^2]/3$

**Table 2.** Selected Bond Distances (Å) and Angles (deg) for *trans*-[$ReO_2$(*trans-R,R*-1,2-DACH)(*trans-S,S*-1,2-DACH)][$BPh_4$]•MeOH•$2H_2O$ (**1**). Atoms generated by symmetry operations are indicated by #1 for cation #1, and #2 for cation #2.[a]

| | | | |
|---|---|---|---|
| | | Bond Distances (Å) | |
| Re(1)-O(1) | 1.760(3) | Re(2)-O(2) | 1.765(3) |
| Re(1)-N(1) | 2.165(3) | Re(2)-N(3) | 2.158(4) |
| Re(1)-N(2) | 2.165(3) | Re(2)-N(4) | 2.175(3) |
| | | Bond Angles (deg) | |
| O(1)-Re(1)-O(1)#1 | 180 | O(2)#2-Re(2)-O(2) | 180 |
| O(1)-Re(1)-N(1)#1 | 89.57(14) | O(2)-Re(2)-N(3) | 89.3(2) |
| O(1)-Re(1)-N(1) | 90.43(14) | O(2)-Re(2)-N(3)#2 | 90.7(2) |
| N(1)#1-Re(1)-N(1) | 180 | N(3)-Re(2)-N(3)#2 | 180 |
| O(1)#1-Re(1)-N(2) | 89.0(2) | O(2)#2-Re(2)-N(4) | 90.59(14) |
| O(1)-Re(1)-N(2) | 91.0(2) | O(2)-Re(2)-N(4) | 89.41(14) |
| N(1)#1-Re(1)-N(2) | 101.10(13) | N(3)-Re(2)-N(4)#2 | 101.2(2) |
| N(1)-Re(1)-N(2) | 78.90(13) | N(3)-Re(2)-N(4) | 78.8(2) |

[a] Symmetry transformations used to generate equivalent atoms: #1 -x+3/2,-y+1/2,-z+1/2; #2 -x,-y,-z

The renal clearance of anionic Tc-99m agents is in general promoted by the presence of an ionized carboxylate group attached to an organic chelating ligand.[23-25] The carboxyl group most likely interacts with the transport receptor. Normally, such anionic Tc-99m agents contain a Tc(V)O core, the oxo ligand of which may also interact with the transport receptor. However, the metal itself is not involved in receptor recognition. In the case of cationic Tc-99m agents, the center of residual positive charge is associated with the metal buried within the *trans* $TcO_2$ core. The metal itself does not directly influence distribution but the oxo ligands, being *trans* to each other, directly compete to donate π electrons into the metal d orbitals. Therefore, the oxo ligands will have more residual charge than the one oxo ligand in the typical renal agent. At present, it is unclear how the axial oxo ligand and also the equatorial ligands affect renal uptake in cationic Tc-99m agents. Our belief that a data base of structural information should help resolve this uncertainty prompted this study.

The structure of the *trans*-[$ReO_2$(*trans-R,R*-1,2-DACH)(*trans-S,S*-1,2-DACH)]$^+$ cation as determined here by X-ray methods provides some useful starting data. The complex is highly symmetrical; consequently only half of the molecule is unique. The molecule has a symmetrical distribution of charge, but on the basis of this structure it is clear that other Tc-99m-DACH isomers must have an unsymmetrical distribution. Thus, overall charge and lipophilicity (similar for all Tc-99m-DACH isomers) exert a greater influence on biodistribution than do the specific structural features of the different Tc-99m-DACH isomers. At present, structural and renal clearance data are limited to relatively few cationic species;[3, 12-15, 26, 27] additional studies combining biodistribution and renal clearance experiments of cationic Tc-99m agents with structure determinations of the Re analogues are needed to establish the structure-distribution relationships required for the development of a clinically useful cationic Tc-99m renal agent.

**ACKNOWLEDGMENTS**

This work was supported by the National Institutes of Health (Grant No. DK38842). We acknowledge useful suggestions from Dr. Patricia A. Marzilli, Emory University Department of Chemistry.

**REFERENCES**

1. Battilana, C., Zhang, H., Olshen, R.A., Wexler, L. , Meyers, B.D. *Am J. Physiol.* **261**, F726-F733 (1991).
2. McNay, J.L., Rosello, S. , Dayton, P.G. *Am J. Physiol.* **230**, 901-906 (1976).
3. Herzog, K.M., Deutsch, E., Deutsch, K., Silberstein, E.B., Sarangarajan, R. , Cacini, W. *J. Nucl. Med.* **33**, 2190-2195 (1992).
4. Preuss, H.G., Massry, S.G., Maher, J.F., Gilliece, M. , Schreiner, G.E. *Nephron* **3**, 265-273 (1966).
5. Bergstrom, J., Bucht, H., Ek, E., Josephson, B., Sundell, H. , Werko, L. *Scan. J. Clin. Lab.* **11**, 361-375 (1959).
6. Brodwall, E.K. *Scan. J. Clin. Lab.* **16**, 12-20 (1964).
7. Boumendil-Podevin, E.F., Podevin, R.A. , Richet, G. *J. Clin. Invest.* **55**, 1142-1152 (1975).
8. Monti, J.P., Gallice, P., Braguer, D., Durand, C., Murisasco, A. , Crevat, A. in *Uremic Toxins* (eds. Ringoir, S., Vanholder, R. & Massry, G.) 223-226 (Plenum Press, New York, 1986).
9. Eshima, D. , Taylor, A., Jr. *Seminars in Nuclear Medicine* **22**, 61-73 (1992).
10. Hansen, L., G., M.L. , Taylor, A. *Q. J. Nucl. Med.* **42**, 280-293 (1998).
11. Peters, L. *Pharmacol. Rev.* **12**, 1-35 (1960).
12. Padhy, A.K., Bomanji, J., Nimmon, C.C., Solanki, K.K., Chaiwatanratan, T. , Britton, K.E. in *Radionuclides in Nephrourology* (eds. O'Reilly, P.H., Taylor, A. & Nally, J.V.) 1-3 (Field & Wood Medical Periodicals, Blue Bell, PA, USA, 1994).
13. Solanki, K.K., Theobald, A.E. , Britton, K.E. *J. Nucl. Med.* **32**, 1102 (abstract) (1991).
14. Solanki, K.K., Bomanji, J., Nimmon, C.C., Carroll, M. , Theobald, A.E. *J. Nucl. Med.* **32**, 1105 (abstract) (1991).
15. Padhy, A.K., Solanki, K.K., Bomanji, J., Chaiwatanaran, T., Nimmon, C. , Britton, K.E. *Nephron* **65**, 294-298 (1993).
16. Taylor, A., Hansen, L., Eshima, D., Malveaux, E., Folks, R., Shattuck, L., Lipowska, M. , Marzilli, L.G. *J. Nucl. Med.* **38**, 821-826 (1997).
17. Fritzberg, A.R., Kasina, S., Eshima, D. , Johnson, D.L. *J. Nucl. Med.* **27**, 111-116 (1986).
18. Taylor, A., Hansen, L. , Marzilli, L.G. submitted
19. Ciani, G.F., D'Alfonso, G., Romiti, P., Sironi, A. , Freni, M. *Inorg. Chim. Acta* **72**, 29-37 (1983).
20. Blessing, R. *Acta Crystallogr.* **A51**, 33-38 (1995).
21. Belanger, S. , Beauchamp, A.L. *Inorg. Chem.* **35**, 7836-7844 (1996).
22. Carey, F.A. , Sundberg, R.J. in *Advanced Organic Chemistry. Part A: Structure and Mechanisms.* 112 (Plenum Press, New York, 1984).
23. Rao, T.N., Adhikesavalu, D., Camerman, A. , Fritzberg, A.R. *J. Am. Chem. Soc.* **112**, 5798-5804 (1990).
24. Hansen, L., Taylor, A., Jr. , Marzilli, L.G. *Met.-Based Drugs* **2**, 105-110 (1995).
25. Marzilli, L.G., Banaszczyk, M.G., Hansen, L., Kuklenyik, Z., Cini, R. , Taylor, A., Jr. *Inorg. Chem.* **33**, 4850-4860 (1994).
26. Sommezoglu, P., Erdil, T.Y., Demir, M., Sayman, H.B., Kabasakal, L., Yardi, O.F., Lzkara, H., Mat, M.C., Solanki, K. , Britton, K.E. *Eur. J. Nucl. Med.* **25**, 1630-1636 (1998).
27. Datseris, I., Boletis, J., Papadakis, E., Baha, S., Leondi, A., Prassopoulos, V., Anagnostopoulos, Z., Vosnides, G. , Kostamis, P. *Eur. J. Nucl. Med.* **22**, 835 (abstract) (1995).

**Received: May 5, 2000 - Accepted: May 23, 2000 -**
**Received in revised camera-ready format: June 8, 2000**

